# Examining the validity and reliability of the Persian version of the MiND‐B questionnaire in ALS patients

**DOI:** 10.1002/brb3.3167

**Published:** 2023-07-24

**Authors:** Reza Rahimzadeh Goradel, Reza Sattarpour, zahra hooshyari, Morvarid Taebi, Afsaneh Ghavampour, Maryam Rashidi Jazani, Payam Sarraf

**Affiliations:** ^1^ Students’ Scientific Research Center, Exceptional Talents Development Center Tehran University of Medical Sciences Tehran Iran; ^2^ Psychiatry and Psychology Research Center Tehran University of Medical Sciences Tehran Iran; ^3^ Tehran medical branch, Islamic Azad University Tehran Iran; ^4^ Iranian center of Neurological Research Neuroscience Institute, Tehran University of Medical Sciences Tehran Iran

**Keywords:** amyotrophic lateral sclerosis, Motor Neuron Disease Behavioral instrument (MiND‐B), Persian

## Abstract

**Background:**

In addition to affecting the nerves and muscles, amyotrophic lateral sclerosis (ALS) disease also affects the behavior and cognition of patients. In this study, we examine the validity and reliability of the Persian version of Motor Neuron Disease Behavioral instrument (MiND‐B) questionnaire to investigate behavioral changes in Persian‐speaking ALS patients.

**Methods:**

Forty‐six Persian‐speaking patients with ALS filled out the MiND‐B questionnaire. Then, the overall scores and each of the domains of this questionnaire were statistically analyzed.

**Results:**

Cronbach's alpha coefficient was calculated .70 for the whole questionnaire. To check the validity of the questionnaire, the correlation of its scores with the Edinburgh Cognitive and Behavioral ALS screen (ECAS‐A) questionnaire was taken, and this correlation was significant (*p* = .038).

**Conclusion:**

The findings of this study show that the Persian version of the MiND‐B questionnaire has the necessary validity and reliability to investigate behavioral changes in Persian‐speaking patients with ALS.

## 1 INTRODUCTION

Amyotrophic lateral sclerosis (ALS) is motor neuron disease (MND) with lesions in both upper and lower motor neurons and can affect either of the facial or limb muscles, presenting with muscle weakness and wasting (Brown & Al‐Chalabi, 2017). Wordwide ALS prevalence is estimated to be 4.1–8.4 per 100,000 people (Andersen et al., 2012; Castro‐Rodríguez et al., 2021) with a median survival of 3–5 years after symptom onset according to different studies (Paulukonis et al., 2015; Zhou et al., 2018). Having no definite cure, ALS patients have to receive palliative care on average 3–5 year course of the disease before there death. Through the course of the disease, 35–40% of patients may demonstrate behavioral and cognitive changes in addition to motor symptoms (Neary et al., 1998). Behavioral impairment in ALS can lower the patients’ quality of life, influence their adherence to the recommended care and treatments, and increase the workload of the caregiver alongside the healthcare providers. This impairment is important to be assessed and monitored so that the physician and the caregiver can be aware of the changes and also can hire the suitable supporting approaches for the patient (Beeldman et al., 2016; Goldstein & Abrahams, 2013).

Behavioral impairment in ALS patients has a wide range, most of which is in the domains of emotion and social behavior, including apathy as the most common, disinhibition, recklessness, and stereotypical behavior (Abe et al., 1997; Neary et al., 2000) Past studies have shown that these disorders can be related to some genotypes. The relationship between C9 or f72 gene mutation and verbal disorders has been investigated in a study. Therefore, symptoms and disorders can be prevented by screening the relevant patients for these types of mutations (Lulé et al., 2020). In addition, other motor neuron diseases like primary lateral sclerosis also show extra motor symptoms such as behavioral disorders (Finegan et al., 2021). Assessing all of these changes requires a valid, short, and sensitive tool that has been developed in recent years. The Motor Neuron Disease Behavioral instrument (MiND‐B) is an English version of questionnaire, which has been proven to be valid and reliable in that language (Mioshi et al., 2014). Our aim in this study was to evaluate the validity and reliability of the Persian translation of MiND‐B to assess behavioral impairment in Persian‐speaking ALS and other MNDs patients so that they could be managed accordingly.

## 2 METHOD

### 2.1 Participants

This study was approved by the Ethical Review Board of Imam Khomeini Hospital and Tehran University of Medical Sciences and carried out according to the Declaration of Helsinki and consisted of internal consistency, reliability, and validity analysis of the Mind‐B questionnaire. All patients diagnosed with ALS who attended the ALS clinic of Imam Khomeini's Neurology Center in the first half of 2022 (*n* = 77) were asked to participate in the study. Patients who were taking drugs affecting cognitive clarity were not included in the study. We took written consent form from all the participants as an inclusion criterion. Finally, 46 patients from both genders with diagnosed ALS completed the translated MiND‐B and Edinburgh Cognitive and Behavioral ALS screen (ECAS‐A) questionnaire.

### 2.2 Instrument

The MiND‐B questionnaire was developed by Mioshi and colleagues to be a simple yet sensitive tool for both identifying and measuring behavioral changes in ALS patients. It is designed to be filled by the caregiver of the patients and consists of nine questions rating patient's behavioral changes on a Likert scale ranging from 0 to 4 and evaluates behavioral changes in three main domains including apathy (three questions, scoring 0–12), disinhibition (four questions, scoring 0–16), and stereotypical behavior (two questions, scoring 0–8) with a total score between 0 and 36 (Mioshi et al., 2014).

ECAS‐A is another questionnaire which was especially designed to screen for cognitive and behavioral impairment in ALS patients and is supposed to be filled by the physician according to the answers of the patients. It evaluates cognitive impairment in domains of language, fluency, executive function, memory, and visuospatial and has a total score of 136. Moreover ECAS‐A involves a part which screens for behavioral changes by which we search for signs of frontotemporal dementia according to checklist (Mojtabavi et al., 2021; Niven et al., 2015; Rascovsky et al., 2011; Snowden et al., 2012).

### 2.3 Questionnaire cross‐cultural adaption

The questionnaire was developed by using the method of Beaton's six‐step cross‐cultural adaption and backward translation to Persian of a previously validated English version of the questionnaire (Beaton et al., 2000).

### 2.4 Statistical analysis

At first, the data were cleaned and the missing data were managed by inserting the average with the aim of improving their effect on the statistical results. For internal consistency and reliability, Cronbach's alpha coefficient and Spearman–Brown coefficient were calculated. Cronbach's alpha coefficient can range from 0 to 1.0 and amounts above .6 are considered satisfactory. Validity was assessed by comparing the results of MiND‐B and ECAS‐A questionnaires about ALS patients’ cognitive condition. All statistical analyses were carried out using IBM Statistical Package for the Social Sciences (SPSS) 26.0. A *p*‐value of .05 was considered statistically significant.

## 3 RESULT

### 3.1 Demographic information

Forty‐six Persian‐speaking patients with ALS diagnosed by a neurologist were included in the study to complete the MiND‐B questionnaire. Table 1 shows the demographic information of the patients, which includes information on age, gender, educational level, dominant hand, and scores of the MiND‐B and ECAS questionnaires. Among them, 31 (67.3%) patients were male and 15 (32.6%) were female. The average age of the patients was 54.9 years (*SD* = 10.3), in the range of 29–47 years (Table 1). The average score of the patients' MiND‐B questionnaire was 19.93 in the range of 9–31 and for the ECAS questionnaire was 76.6 and in the range of 40–110.

**TABLE 1:** Demographic information.

	Demographic variables	*N*	Valid percentage	Mean (*SD*)
Age(year)		46	–	54.93 (10.3)
Gender	Male	31	67.4	–
	Female	15	32.6	
Educational level	Below diploma	20	43.5	–
	Diploma	13	28.3	
	Bachelor	10	21.7	
	Master	3	6.5	
Dominant hand	Right	46	100	
	Left	0	0	
ALS‐FRS		46	100	31.95 (10.43)
ECAS‐A		23	50	78.21 (23.50)
MiND‐B scores		46	100	19.93 (5.56)

Abbreviations: ECAS‐A, Edinburgh Cognitive and Behavioral ALS screen; MiND‐B, Motor Neuron Disease Behavioral instrument. ALS‐FRS, Amyotrophic lateral sclerosis functional rating score.

### 3.2 Reliability and internal consistency

The other statistic indices for the patient's MiND‐B questionnaire is given in Table 2 separately for each question. Cronbach's alpha coefficient was calculated as .70 for the whole questionnaire. Cronbach's alpha coefficient was also calculated for two split of the questionnaire. The value was calculated as .702 for Part 1 and .495 for Part 2. The Spearman–Brown coefficient as an internal consistency index was calculated as .517.

**TABLE 2:** MiND‐B questionnaire statistical description. Item‐total correlation and validity of Cronbach's alpha, if one item was deleted.

Questionnaire	*N*	Mean (*SD*)	Corrected item‐total correlation	Cronbach's alpha if item deleted
1. He/she has temper outbursts.	46	2.40 (1.09)	.348	.677
2. He/she is uncooperative when asked to do something.	46	2.02 (1.19)	.440	.658
3. He/she makes tactless or suggestive remarks.	46	1.69 (1.04)	.413	.666
4. He/she acts impulsively without Thinking.	46	1.77 (1.00)	.447	.660
5. He/she shows less enthusiasm for their usual interests.	46	2.28 (1.36)	.517	.639
6. He/she shows little interest in doing new things.	46	2.73 (1.35)	.213	.710
7. He/she fails to maintain motivation to keep in contact with friends or family.	46	1.75 (1.12)	.455	.656
8. He/she is rigid in their ideas and Opinions.	46	3.57 (0.81)	.131	.708
9. He/she repeatedly uses the same expression or catchphrase.	46	1.69 (1.17)	.392	.669

Abbreviations: MiND‐B, Motor Neuron Disease Behavioral instrument ; *SD*, standard deviation.

### 3.3 Validity

To evaluate the validity of the MiND‐B questionnaire, the correlation was calculated between the scores of this questionnaire and the scores related to cognitive changes of the ECAS‐A questionnaire. The correlation coefficient of MiND‐B scores with ECAS‐A scores was −.435 (*p* = .038) as an index of divergent validity. The correlation coefficients of MiND B scores and ECAS‐A scores of patients' cognitive domains are reported in Table 3.

**TABLE 3:** Correlation between MiND‐B scores and ECAS‐A scores by cognitive domains.

		Language	Linguistic psychology	Executive function	memory	visuospatial	ECAS‐A scores
MiND‐B scores	*R*‐value	−.437	−.243	−.475	−.248	−.068	−.435
	*p‐*value	.016	.223	.014	.203	.719	.038
	*N*	30	27	26	28	30	23

Abbreviations: ECAS‐A, Edinburgh Cognitive and Behavioral ALS screen; MiND‐B, Motor Neuron Disease Behavioral instrument.

## 4 DISCUSSION

Due to the high prevalence of behavioral changes in patients with ALS, the study of these changes in patients and their management can increase the quality of life of the patients and their caregivers, in addition to helping the healthcare providers to facilitate the treatment process.

Previous studies have examined the validity and reliability of the English version of MiND‐B. In addition to an original study, two systematic review studies which examined various behavioral impairment measuring tools showed that the MiND‐B questionnaire has the required validity and reliability to assess behavioral changes in ALS patients (Gosselt et al., 2020; Mioshi et al., 2014; Simon & Goldstein, 2019). In this study, we examined the validity and reliability of the Persian version of this questionnaire.

Based on the reported statistical findings, the internal consistency and Cronbach's alpha coefficient of the Persian version of the MiND‐B questionnaire are .698, which shows that this questionnaire has good internal consistency and reliability for research uses (Bland & Altman, 1997). The Spearman–Brown coefficient of .517 also confirms this finding. In addition, according to statistical findings, omitting any of the questions can increase Cronbach's alpha coefficient and increase the internal consistency of the questionnaire. Of course, by removing number 6 and 8 questions, the largest increase in Cronbach's alpha coefficient occurs. These two items question the interest in doing new works and the rigidity of beliefs. Moreover, the statistical results demonstrate that the first part of the questionnaire, including the first five questions, has a larger Cronbach's alpha coefficient than the later four questions. An explanation to this difference between the two parts can be that in the first part the questions address changes that happen more simultaneously while in the second part the questions address more discrete behaviors. Furthermore, the corrected item‐total correlation indicates that this value is high for most questions which shows that the majority of questions are well‐discriminated and that the only question with a low value is question number 8 (Table 2).

To evaluate the validity of the MiND‐B questionnaire, in this study, we measured the correlation between the MiND‐B scores and the scores of the Persian version of the ECAS‐A questionnaire. The ECAS‐A questionnaire is used to assess cognitive changes in ALS patients and has different cognitive domains including memory, language, and executive function. Our statistical findings show that there is a negative correlation between the scores of these two questionnaires with a coefficient of −.435 while this correlation is significant (*p* = .038). The negative correlation is due to the fact that either a lower ECAS‐A score or a higher MiND‐B score both are related to a more severe impairment. Therefore, the negative correlation indicates the direction of changes in the scores of these two instruments. So this result demonstrates that this questionnaire can be used to monitor the behavioral changes of ALS patients. In addition, to examine the degree of correlation by cognitive domains, there is a significant correlation between MiND‐B scores as an indication of behavioral changes with the language and executive function domains addressed in ECAS‐A (Table 3). Of course, it should be noted that the overall correlation of ECAS‐A with MiND‐B is significant and indicates the alignment of changes and validity of MiND‐B for research uses in ALS patients.

The findings of other studies also confirm the results of our study. Two systematic reviews were performed on the subject of validity and reliability of various behavioral disorders questionnaires showed the MiND‐B questionnaire has good validity and reliability, and this questionnaire can be used to assess behavioral changes in patients with ALS (Gosselt et al., 2020; Simon & Goldstein, 2019).

Our team also had limitations in conducting this study. The most important of these limitations was the inability of a wide range of patients to speak and communicate with family and the study team which challenged the interview process with the patient and his companions. Another limitation was our lack of access to many other behavioral and cognitive tests, the reason being that very few studies have been conducted on ALS in our country, and many of these tests have not been validated in Persian. Also, we did not have access to the genetic data of the patients. And an important limitation was that we lost the patients who are in the lower stages of ALS due to the lack of proper diagnosis in other centers and their lack of referral to our referral center. Considering all these limitations, we did our best to reduce the effects of these limitations on the results of our study.

As mentioned, the Persian version of the MiND‐B questionnaire is suitable and acceptable for measuring behavioral changes in ALS patients with validity, reliability, and, internal consistency and this questionnaire can be used for research affairs to assess the behavioral changes of Persian‐speaking patients with ALS.

### PEER REVIEW

The peer review history for this article is available at https://publons.com/publon/10.1002/brb3.3167.

## Data Availability

The data that support the findings of this study are available from the corresponding author upon reasonable request.
